# High Prevalence of Multistability of Rest States and Bursting in a Database of a Model Neuron

**DOI:** 10.1371/journal.pcbi.1002930

**Published:** 2013-03-07

**Authors:** Bóris Marin, William H. Barnett, Anca Doloc-Mihu, Ronald L. Calabrese, Gennady S. Cymbalyuk

**Affiliations:** 1 Instituto de Física, Universidade de São Paulo, São Paulo, São Paulo, Brazil; 2 Neuroscience Institute, Georgia State University, Atlanta, Georgia, United States of America; 3 Department of Biology, Emory University, Atlanta, Georgia, United States of America; University of Pittsburgh, United States of America

## Abstract

Flexibility in neuronal circuits has its roots in the dynamical richness of their neurons. Depending on their membrane properties single neurons can produce a plethora of activity regimes including silence, spiking and bursting. What is less appreciated is that these regimes can coexist with each other so that a transient stimulus can cause persistent change in the activity of a given neuron. Such multistability of the neuronal dynamics has been shown in a variety of neurons under different modulatory conditions. It can play either a functional role or present a substrate for dynamical diseases. We considered a database of an isolated leech heart interneuron model that can display silent, tonic spiking and bursting regimes. We analyzed only the cases of endogenous bursters producing functional half-center oscillators (HCOs). Using a one parameter (the leak conductance (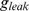)) bifurcation analysis, we extended the database to include silent regimes (stationary states) and systematically classified cases for the coexistence of silent and bursting regimes. We showed that different cases could exhibit two stable depolarized stationary states and two hyperpolarized stationary states in addition to various spiking and bursting regimes. We analyzed all cases of endogenous bursters and found that 18% of the cases were multistable, exhibiting coexistences of stationary states and bursting. Moreover, 91% of the cases exhibited multistability in some range of 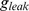. We also explored HCOs built of multistable neuron cases with coexisting stationary states and a bursting regime. In 96% of cases analyzed, the HCOs resumed normal alternating bursting after one of the neurons was reset to a stationary state, proving themselves robust against this perturbation.

## Author Summary

It is often not appreciated that different activity regimes can coexist with each other in a given neuron so that a transient stimulus can cause a persistent change of activity. Such multistability of the neuronal dynamics has in fact been shown in a variety of neurons and can play either a functional role or present a substrate for neurological diseases. We explored the propensity for multistability in a database of a leech heart interneuron model, testing each case (parameter set) in a database for multistability. We found a large proportion of multistable cases, especially the coexistence of silent and bursting regimes. This was a surprising result, since these cells pace the heartbeat of the leech, and the coexistence of silence and bursting could disrupt the functional pattern, threatening the viability of the leech. Analysis of networks of mutually inhibitory multistable neurons, however, showed robustness in maintaining functional activity, suggesting that the mutually inhibitory coupling can act as a protective mechanism against failures induced by multistability.

## Introduction

Recent studies of neuronal networks of identifiable neurons have shown that the same neuron type can significantly vary in membrane properties from animal to animal. The biophysical characteristics of the single neurons performing the same task can be orders-of-magnitude different [1]–[4]. This fact testifies to the great flexibility and robustness demonstrated by nervous systems. It is also captured by mathematical models analyzed with brute-force databases. With a database a population of models is considered so that those parameter sets (cases) which satisfy constraints derived from experimental data are identified as functional. Thus, following this approach, we obtained a set of cases producing functional activity although the underlying ionic current compositions were different. The apparent simplicity of the product of the brute-force database approach is moderated by complications posed by multistability.

Single neurons can produce a plethora of regimes of activity including silent, spiking and bursting regimes depending on their membrane properties. What is less appreciated is that these regimes can coexist with each other. Multistability has been reported for different neurons in a number of experimental and modeling studies [5]–[18]. It can play either a functional role or present a substrate for dynamical diseases. A comprehensive database of a neuronal model should attempt to describe all possible observable regimes of activity to assess the functionality of each case of the model.

Neuronal models exhibit a variety of activities depending on the set of parameters chosen. Parameter set databases of computational models are powerful tools used to understand how different components of neuronal dynamics interact to produce functional activity. Brute-force database approaches classify these dependencies and infer the roles played by intrinsic membrane and synaptic currents in the normal and pathological dynamics of the neuronal system of interest. These applications have proven their effectiveness in finding suitable parameter regimes that fit experimental measurements and recorded activities, and have shown that a large variety of the parameter regimes can satisfy experimental constraints [2], [19]–[25]. Here we show that it is feasible to expand such a database by appending information about stable and unstable stationary states. To obtain this information, we applied techniques from the bifurcation theory. These techniques allowed us to systematically reveal cases of multistability of bursting and stationary states.

Doloc-Mihu and Calabrese [25] have built a database for a model of an isolated leech heart interneuron and a model of the half-center oscillator consisting of a reciprocally inhibitory pair of interneurons (HCO). An individual leech heart interneuron is represented as a single isopotential electrical compartment with eight Hodgkin-Huxley type intrinsic membrane conductances [26]. In addition, the model of the HCO includes two types of inhibitory synaptic currents, spike mediated and graded [26]. By varying a set of 8 key parameters (the leak reversal potential and maximal conductances of synaptic and several membrane currents) in all combinations possible (brute-force approach) the authors of [25] systematically explored the parameter space and analyzed more than 10 million simulations (cases) of the model.

In this study, we wanted to assess how prevalent multistability of bursting and silent regimes is in a population of functional cases of the leech heart interneuron. Application of the theory of dynamical systems allows mapping the transitions between the regimes by using bifurcation diagrams [10], [17], [18], [27]. Through the use of numerical continuation of stationary states, we incorporated information about stable and unstable stationary states into the database developed in [25]. This novel approach does not resort to direct integration of differential equations, circumventing the arbitrariness in the choice of initial conditions, besides being less computationally demanding. Our methodology allowed us to systematically reveal cases of multistability in the database, and it turned out that the number of such cases is surprisingly large. Coexistence of silent regimes with functional bursting regime of leech heart interneurons poses a threat to viability of the animal since they pace the heartbeat. We also investigated how alteration of leak current and network interactions could resolve this potential problem.

## Methods

### Model and database

Doloc-Mihu and Calabrese [25] built an extensive database of a model of a half-center oscillator (HCO). This model was developed to represent dynamics of the elemental oscillator which produces the basic rhythm of the central pattern generator controlling heartbeat of the medicinal leech [26]. It consists of two reciprocally inhibitory identical neurons. The canonical model replicates the electrical activity of the oscillator interneurons of the leech heartbeat central pattern generator (CPG) under a variety of experimental conditions [26]. Doloc-Mihu and Calabrese [25] analyzed activity regimes of the HCO model implemented in *Genesis*2.3, a software for simulation of neuronal dynamics [28]. Each individual leech heart interneuron (HN) was modeled as a single isopotential electrical compartment with Hodgkin and Huxley type intrinsic membrane currents. It has 8 voltage-gated currents: 1) I_Na_- fast Na^+^ current, 2) I_P_ - persistent Na^+^ current, 3) I_CaF_- rapidly inactivating low-threshold Ca^++^ current, 4) I_CaS_ - slowly inactivating low-threshold Ca^++^ current, 5) I_h_- hyperpolarization-activated cation current, 6) I_K1_ - delayed rectifier-like K^+^ current, 7) I_K2_ - a persistent K^+^ current, and 8) I_KA_ - a fast transient K^+^ current. The model also included two types of inhibitory synaptic currents between the two interneurons: graded I_SynG_, and spike-mediated I_SynS_. The differential equations describing the model are given in the appendix of Hill et al. [26].

Doloc-Mihu and Calabrese [25] used a brute-force approach to explore the parameter space of the HCO model, by systematically varying a set of eight key parameters: the leak conductance and reversal potential, the maximal conductances of the spike-mediated and graded synaptic currents, 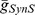 and 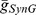, and the maximal conductances of I_P_, I_CaS_, I_h_, and I_K2_. Five distinct values were used for the leak reversal potential (−70 mV, −65 mV, −60 mV, −55 mV, −50 mV) and eight values were used for the maximal conductances, which were set to 0%, 25%, 50%, 75%, 100%, 125%, 150%, and 175% of the canonical values described in Hill et al. [26]. All possible combinations of the values of the varied parameters were tested. This brute-force approach generated a grid in the parameter space of the HCO model consisting of 10,321,920 cases, which had at least one of the two types of synaptic currents present (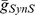
≠0 nS and/or 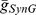
≠0 nS). A special set of the database represents decoupled neurons (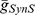 = 0 nS and 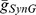 = 0 nS). This set consisted of 163,840 cases of the *isolated neuron model*. All simulations of HCOs were started from the same initial conditions such that one of the two cells was firing while the other was hyperpolarized. These initial conditions were picked as a point on a bursting trajectory of the canonical HCO, obtained as the last point of an activity trace generated by integrating the canonical model for 200 s. For each case of the isolated neuron model, two trajectories were generated starting from the two different initial states described for the pair of neurons in the HCO model.

Trajectories obtained for each case were analyzed and a plethora of different regimes were found and classified as subsets of the database [25]. The classification of the trajectories is mostly based on the analysis of spike times. Spikes were detected by finding the maximum value of the membrane potential above a threshold of −10 mV. Any trace that didn't contain spikes was classified as silent (sub-threshold oscillations would be considered as a silent regime). Time series with all inter-spike intervals (ISI) smaller than 1 s were classified as tonic spiking. If at least one ISI was larger than 1 s, bursting descriptive statistics were calculated determining interburst interval, burst duration, and period of bursting. If the coefficient of variation of bursting period was higher than 5%, the trace was classified as irregular. Furthermore, Doloc-Mihu and Calabrese [25] defined HCO models as functional if both cells exhibited regular bursting activity with a small variability of the burst period (coefficient of variation of the bursting period smaller than 5%) and relative phase in the range 0.45–0.55 (balanced activity).

**Figure 1:**
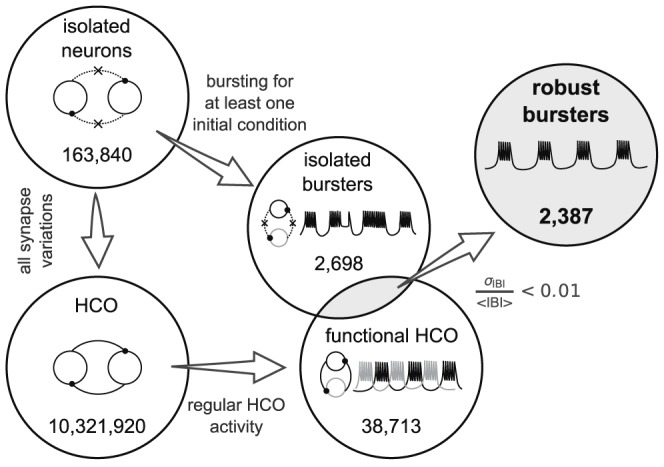
Diagrammatic illustration of the extraction of the robust burster subset from the leech heart interneuron database. The database describes the model of the isolated leech heart interneuron (163,840 cases) and the model of the half-center oscillator (10,321,920 cases). Among the cases representing the isolated interneurons there are a few which produce bursting activity (2,698 cases); and there are a small number of cases of the model of half-center oscillators which produce functional activity (38,713 cases). The subset of interest (2,387 robust bursters) was obtained as the intersection of these sets with the additional requirement of producing steady endogenous bursting activity (coefficient of variation of the interburst interval smaller than 10%).

We focused our analysis on a subset of the cases in the database which conformed to a number of constraints on the activity. These cases were classified as the *robust bursters* ([25], Figure 1). When disconnected (i.e., as isolated neurons), they exhibited robust bursting activity for at least one of the two initial conditions. This bursting activity was not plateau-like or *irregular*. The coefficient of variation of the period was less than 5%. While connected into a HCO, they produced a functional alternating bursting pattern for at least some values of synaptic conductances. In addition, the coefficient of variation of the interburst interval was smaller than 10% (formulae Figure 1). The database subset of such isolated neuron cases (*robust bursters*) had a total number of 2,387.

### Computational details

In this study, numerical integration of the model equation implemented in C was performed with an implicit Runge-Kutta method of 5-th order, Radau IIa [29], suitable for stiff systems of ordinary differential equations. The relative error tolerances were 10^−9^ for each state variable, and the absolute tolerance was 10^−12^. Unless otherwise mentioned, the trajectories were integrated for 400 s (model time); the initial 200 s of activity were discarded to remove the transient part of the trajectory, and the last 200 s were analyzed.

### Continuation of stationary states

**Figure 2:**
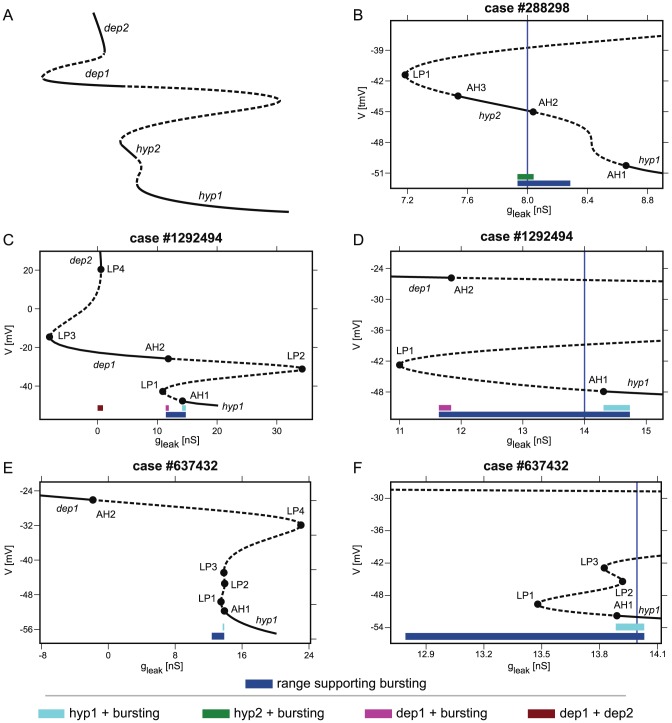
One parameter bifurcation diagrams of stationary states of the robust burster cases from the leech heart interneuron database. The bifurcation parameter is the conductance of the leak current, 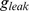. The solid and dashed intervals of a bifurcation curve denote stable and unstable stationary state branches, respectively. Labeled points in panels B–F indicate bifurcations of stationary states: LP and AH stand for the fold and Andronov-Hopf bifurcations, respectively. The navy blue bars indicate the ranges of 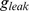 values supporting attracting bursting regimes, while the colored bars above them denote ranges supporting the coexistence of distinct attracting regimes. The color code of the bars is described in the key and is consistent between figures (Figures 2,4,6). Panels D, and F are magnifications of the diagrams around the range supporting bursting in panels C and E, respectively. The vertical navy blue lines in panels B, D, and F and Figure 4B indicate the original 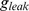 value for the case in the database. A: A stylized bifurcation diagram illustrating the naming convention for the intervals on the branches where stationary states are stable: *hyp1*, *hyp2*, *dep1*, and *dep2*. B: Magnification of the bifurcation diagram for case #288298 that displays a stable interval *hyp2* on the middle branch delimited by two Andronov-Hopf bifurcations, AH2 and AH3. The green bar denotes a 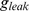 range supporting bursting and the *hyp2* stationary state. C and D: In the case #1292494 there are three stable stationary state intervals. C: The red brown bar indicates a range of 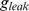 values supporting the coexistence of *dep1* and *dep2* stationary states. The Andronov-Hopf bifurcation (AH2) and the fold bifurcation LP3 determine the *dep1* interval. The fold bifurcation (LP4) defines the right border of the *dep2* interval. The left border of the bistability range is 0 nS. C and D: The magenta bar marks coexistence of *dep1* and bursting. The bar is limited by the range supporting bursting on the left side and by Andronov-Hopf bifurcation AH2 on the right side. The cyan bar indicates coexistence of *hyp1* and bursting between the Andronov-Hopf Bifurcation (AH1) and the border where bursting disappears. E and F: A case where the original 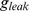 value in the database falls into a range of bistability of bursting and *hyp1* stationary state (cyan bar). The bistability range is defined by the Andronov-Hopf bifurcation (AH1) and the right border where bursting disappears.

For each robust burster case, we performed a single parameter bifurcation analysis of the stationary states. The leak current conductance 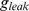 was used as the controlling parameter. The continuation was initiated from the stable hyperpolarized stationary state obtained by direct integration of the system with a large 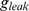 value (20 nS). The coordinates of all codimension 1 bifurcations of stationary states (saddle-node and Andronov-Hopf bifurcations) as well as the stability of each stationary state in the curve were recorded (Figure 2). Numerical continuation of stationary states was performed with the PyDSTool Python package [30], using 4,000 control parameter steps, with minimum and maximum step sizes of 0.02 nS and 10^−12^ nS respectively.

### Ranges of 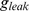 supporting robust bursting activity

We determined the ranges of 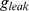 for which the robust bursters exhibited robust bursting activity. Starting from the original 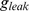 value from the database, we iteratively incremented 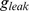 by 1 nS steps and integrated the model, until a transition from bursting to silence, tonic spiking, or irregular bursting was detected. Once this transition was found, the initial conditions were reset to the coordinates of the endpoint of the last accepted bursting trajectory, and the process repeated with a tenfold smaller 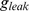 step size to achieve a certain precision of the critical parameter value. This procedure was iterated until the precision of 10^−4^ nS had been achieved. Then, the whole procedure was repeated with negative steps, to determine the boundary of robust bursting towards smaller 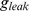 values. Thus a range of 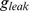 values was determined for which the robust bursting regime was observed. All analysis and auxiliary scripts were developed in-house, using Python 2.6 along with the SciPy package [31], and were run on an Intel Core i7 platform cluster. The calculation of steady state curves (numerical continuation) for 2,387 robust bursters took approximately 2.5 cpu days, while calculating the ranges of 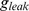 supporting bursting (direct integration) took approximately 3.5 cpu days.

### Ranges of 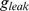 supporting multistability

The ranges of 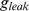 supporting the coexistence of attracting regimes were determined by checking for overlaps of the 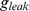 ranges supporting robust bursting activity and the ranges supporting stable stationary states, obtained from the continuation curves. Similarly, ranges supporting multistability of stationary states were determined by finding the overlaps of the corresponding ranges.

### Upgrading the database with information on stationary states and multistability

For building the database extension concerning the stationary states, we used home-written Java scripts (Java 1.5). We used the Java language for several reasons: to have scripts that can be run as is on different operating systems, to be able to query a large table (millions of records), and to be consistent with the previous work on the HCO model [25]. The database was built as two standalone database tables using MySQL (www.mysql.com). Now, for the cases considered in this article one database table has entries describing each determined stationary state by its coordinates in the state space and information on stability. This database table also has entries describing types of multistability determined. The second database table contains all the bifurcation values of the bifurcations determined in the first table. This information was recorded into the tables by using the same identification (unique) number as it had in the original HCO/single neuron database. In this way, one could query the three databases simultaneously, or could just work only with the latter.

### Half-center oscillator analysis

To determine the effects of multistability on neurons in a half-center oscillator, we used the HCO model composed of two identical neurons connected through both spike mediated and graded synaptic currents [10], [26]. This is the simplest network that retains the topology and synaptic currents observed in the leech heartbeat central pattern generator, and allows direct checking of whether the presence of stable stationary states can disrupt rhythmic pattern generation. The simulation protocol consisted of setting the maximal synaptic conductances 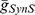 and 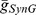 to 150 nS and 30 nS respectively, and then of integrating the HCO model for 100 s. Then one of the cells had its initial conditions reset (not clamped) to a stable stationary state (two possibilities in the case of tristable cells), and the synaptic interactions were removed for 5 s, by setting their maximum conductances 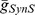 and 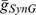 to 0 nS. After these 5 s, the synaptic currents were restored and the system was integrated for additional 100 s. Bursting activity was analyzed separately for both the initial and final intervals.

## Results

### Analysis of robust bursting cases – Continuation of stationary states

Application of bifurcation analysis allowed us to obtain information about stationary states for each case considered from the database. These results are not sensitive to the choice of the initial conditions. We used single parameter bifurcation diagrams: the stationary state curves. It is difficult to obtain such steady state information by direct integration from random initial conditions, as a brute force gridding of the model's high dimensional state space would involve prohibitive computational power requirements. We chose the leak current conductance 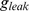 as the continuation parameter because it is present in vast majority of biophysically meaningful models of neurons. In our previous work [10], [17], [26], we have shown its importance in shaping multistable behavior in leech heart neuron models.

In Figure 2A, we sketch the naming convention for the intervals of the bifurcation parameter supporting stable stationary states on the branches of the stationary state curve. We classified stationary states as either hyperpolarized (*hyp*) or depolarized (*dep*) ones, if the membrane potential was negative or positive to −35 mV, respectively. For some cases we found that there could be more than one interval of each type. We numbered them in a sequence so that the *hyp1* interval includes the first point of the curve. For example, the case # 288298 exhibits stable *hyp2* stationary state (Figure 2B). The approach was based on the assumption that for any case of the database, making the leak conductance sufficiently large will cause the model to exhibit a stable stationary state. For the cases considered (robust bursters) it was sufficient to set 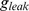 = 20 nS. To establish the first point (*hyp1*) on the stationary state curve we integrated the model with this large value of 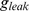. We used bifurcation analysis software to follow this stationary state as the parameter 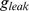 was decreased. In the vast majority of cases, the *hyp1* state lost stability at some smaller value of 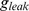 via an Andronov-Hopf bifurcation (AH1). After that the stationary state was continued on the curve as an unstable one. We numbered *hyp* and *dep* states in a sequence, following the curve.

As an example, let us consider the bifurcation diagram of case #1292494 (Figure 2C,D). We continued the stable stationary state exhibited at 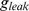 = 20 nS. It lost stability at an Andronov-Hopf bifurcation (AH1). The bifurcation analysis software allowed us to continue the now unstable stationary state. The interval *hyp1* includes only stable stationary states. Thus, it is located on the diagram to the right from AH1 bifurcation value of 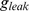. As we traced the stationary state further, it disappeared at a fold bifurcation marked by LP1. At this bifurcation point two unstable stationary states met and disappeared. On the diagram, the curve turned at this bifurcation value, and proceeded to the right, towards larger values of 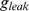. At the value of 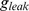 marked on the diagram by LP2, another fold bifurcation occurred and the curve of the stationary state made a second turn. The curve still represented unstable stationary states. Notice that, after the second Andronov-Hopf bifurcation (AH2) on the curve, the stationary state curve reached a stable interval (*dep1*). A stable stationary state from this interval represents a neuron with excitability block. The stable stationary states persisted even after 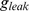 turned negative until it coalesced with an unstable stationary state at a saddle-node (LP3) bifurcation. Tracing the stationary states after 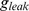
turned negative does not have biophysical meaning, but, interestingly, this unstable branch reached back to the positive 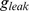 values, where it coalesced with a new stable stationary state branch (*dep2*) at yet another fold bifurcation (LP4). This highly depolarized stationary state has never been observed in models or experimental studies of the leech heart interneurons. A simple analysis of this bifurcation diagram reveals overlap of *dep1* and *dep2* intervals, determining a range of multistability supporting both stable stationary states (Figure 2C).

**Figure 3:**
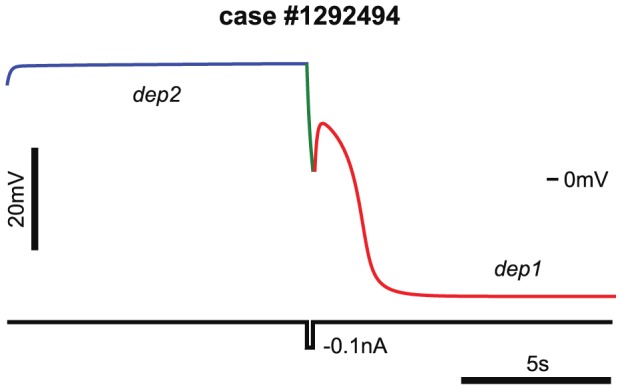
A switch between *dep2* and *dep1* stationary states triggered by a short hyperpolarizing pulse of current. These states correspond to two states of excitability block. The case (#1292494) leak conductance in the database is 14 nS. The value used, 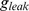 = 0.5783 nS, is taken from the interval of coexistence of the *dep1* and *dep2* branches of stationary states, denoted by a red brown bar in Figure 2C. The model was started near the *dep2* stationary state (blue line), and was perturbed with a square pulse of current after 10 s (green line). The amplitude and the duration of the pulse were −0.1 nA and 0.2 s, correspondingly. The red line depicts transition of the neuron into *dep1* state.

The multistability of two excitability blocks with different values of the rest potential was demonstrated by a perturbation which triggers a switch from one regime to the other. First, by using information from the stationary state curves in Figure 2C, we chose a 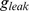
value from the range supporting both stationary states (denoted by a red brown bar in Figure 2C). The bifurcation curve provides the initial conditions corresponding to the *dep2* stationary state. For the demonstration, we selected initial conditions in a vicinity of the actual *dep2* stationary state. A switch from the *dep2* to the *dep1* state was induced by a brief pulse of hyperpolarizing current (Figure 3).

There was also a case that presented a 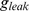 range supporting stable stationary states in the middle branch (*hyp2*) of the continuation curve, as seen in the expanded section of the bifurcation diagram for case #288298 displayed in Figure 2B. The interval of *hyp2* is delimited by two Andronov-Hopf bifurcations. If the system is integrated from initial conditions close to this region, it will give rise to subthreshold oscillations damped towards the *hyp2* stationary state.

### Multistability of regimes

Our methodology also allowed us to systematically detect multistability, when the ranges of 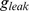 values corresponding to stable stationary states and to robust bursting activity overlapped. For this analysis, the cases from the database which differ only in 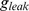 belong to the same bifurcation diagram and are treated as one case thus giving rise to 2,223 cases of *unique robust bursters* instead of the original 2,387 cases. We considered five distinct regimes: the hyperpolarized stable stationary states *hyp1* and *hyp2*, the depolarized ones *dep1* and *dep2*, and bursting (Figure 2A), and screened each case for all possible coexistences of these regimes.

**Figure 4:**
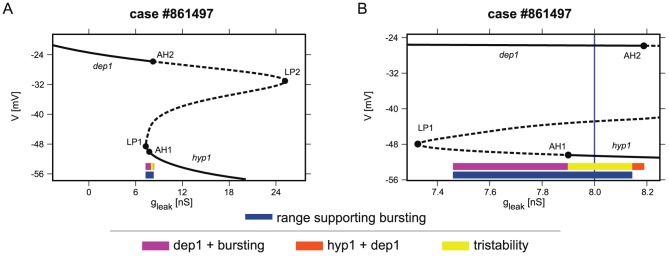
One parameter bifurcation diagram of stationary states of the case #861497 with the marked range supporting bursting. The bifurcation diagram (A) illustrates tristability. The inset (B) is focused on the range of coexistence of hyp1 and dep1 stationary states and bursting regime. The range of 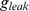, where the bursting regime is an attractor, is indicated by a navy blue bar. The magenta bar marks the range of coexistence of the bursting regime and *dep1* stationary state. The 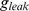 range supporting tristability, where the neuron can exhibit either the bursting regime, *hyp1* stationary state, or *dep1* stationary state is marked by the yellow bar. The range supporting coexistence of *hyp1* and *dep1* states without a bursting regime is marked by an orange bar. Notice that the original 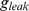 value (8 nS) from the database (indicated by the blue vertical line) belongs to the range supporting tristability.

In the bifurcation diagram for case #1292494 (Figure 2C,D), we can locate three 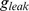 ranges supporting multistability, denoted by colored bars below the continuation curve: the red brown bar - coexistence of *dep2* and *dep1* stationary states, the magenta bar -*dep1* and bursting, and the cyan bar -*hyp1* and bursting. Multistability is also prominently present in case #861497, as can be seen in the bifurcation diagram shown in Figure 4. In the diagram, the yellow bar indicates the coexistence of three regimes, bursting, *hyp1* and *dep1* stationary states (tristability), while the orange bar corresponds to the coexistence of *hyp1* and *dep1* stationary states (bistability) and the magenta bar locates the coexistence of bursting and the *dep1* stationary state (bistability).

**Figure 5:**
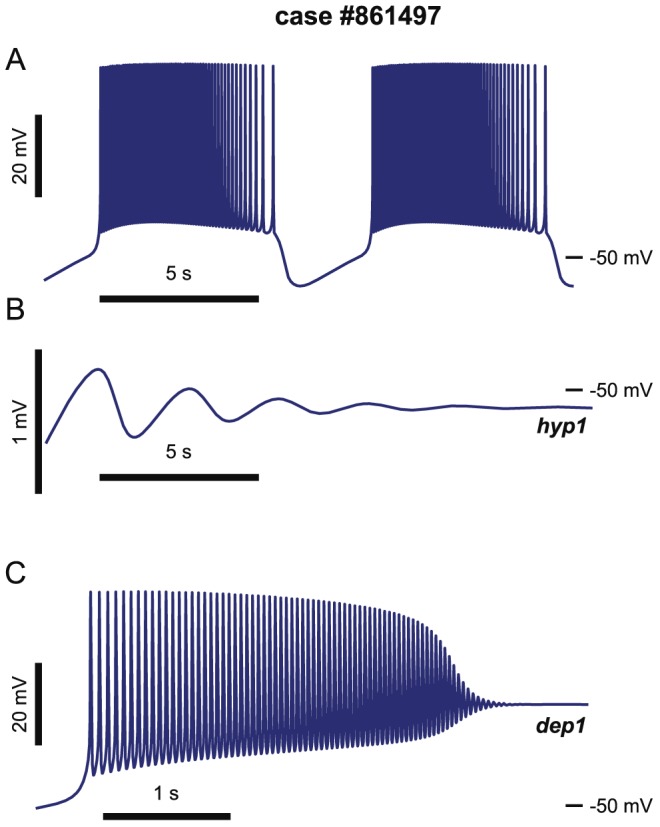
Three different regimes of steady state activity are attractors of a tristable model – two distinct silent states coexist with bursting. On the bifurcation diagram of case #861497 there is a range of 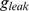 values supporting tristability of bursting, *dep1*, and *hyp1* regimes (Figure 4B). Here, we used the original database value, 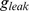 = 8.0 nS. A: the neuron exhibits steady bursting. B: the neuron exhibits slow damped oscillations towards the *hyp1* stationary state. C: the neuron transiently fires spikes damping onto the *dep1* stationary state.

We illustrated tristability detected in Figure 4 by integrating case #861497 from three different sets of initial conditions (Figure 5). The first set of initial conditions led to bursting (Figure 5A). The second set of initial conditions led to slow oscillations damped into the *hyp1* stationary state (Figure 5B), and the third set of initial conditions produced damped spiking oscillations towards the *dep1* stationary state (Figure 5C).

**Figure 6:**
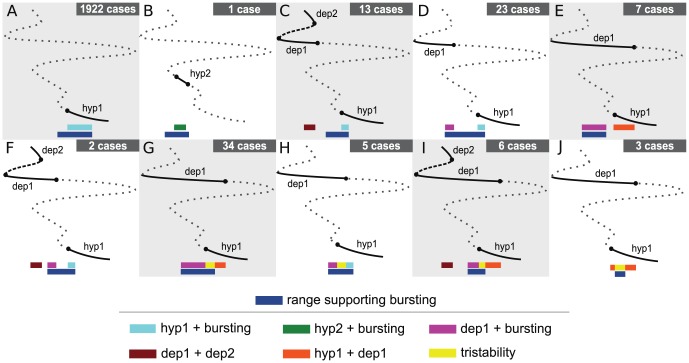
A classification scheme for the cases with multistability. The bifurcation diagrams describe stationary states branches as the conductance of the leak current is varied and have the ranges supporting bursting marked. There are 10 multistability arrangements classified here: (A) bursting and the *hyp1*; (B) bursting with *hyp2*; (C) a range supporting *hyp1* and bursting along with a range supporting *dep1* and *dep2*; (D) a range of bistability of *hyp1* and bursting along with a range of bistability of *dep1* and bursting; (E) one range of coexisting *hyp1* and *dep1* and one range of coexisting *dep1* and bursting; (F) three ranges of coexistence of exactly two regimes: (1) *dep1* and *dep2*, (2) *dep1* and bursting, and (3) *hyp1* and bursting; (G) one range of tristability along with a range of coexistence of *hyp1* and *dep1* and a range of coexistence of *dep1* and bursting; (H) one range of tristability along with a range of bistability of *hyp1* and bursting and a range of bistability of *dep1* and bursting; (I) Four ranges of multistability: a range of tristability along with a range of bistability of *hyp1* and *dep1*, a range of bistability of *dep1* and bursting, and a range of bistability of *dep1* and *dep2*; (J) two ranges of bistability of *hyp1* and *dep1* in addition to a range supporting tristability. The solid (dotted) curves represent stable (unstable) branches. The navy blue bar underlies the range supporting bursting activity. The other colored bars mark different types of multistability as indicated in a key.

Applying this procedure to the whole *unique robust burster* subset (2,223 cases), we obtained the following five multistability scenarios upon variations of 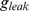 (Figure 6):

1. One range of multistability, supporting exactly two attracting regimes. Considering the cases with only one overlapping range, the vast majority showed coexistence of bursting and the *hyp1* stationary state. Of the 2,223 unique robust bursting cases, 1,922 (86%) showed this type of bistability under variation of 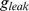 (Figure 6A). We found only one case of co-existence of bursting with *hyp2* (case #288298 of Figure 2B, Figure 6B).
2. Two ranges of multistability, each one supporting exactly two attracting regimes. We also found cases with exactly two ranges supporting exactly two attracting regimes. 13 cases showed one range of coexistence of the *hyp1* stationary state and bursting, along with another range of bistability of the two depolarized stationary states, *dep1* and *dep2* (Figure 6C). 23 cases showed one range of coexistence of the *hyp1* stationary state and bursting, along with one range of coexistence of the *dep1* stationary state and bursting (Figure 6D). 7 cases exhibited one range of coexisting *hyp1* and *dep1* stationary states and one range of coexistence of the *dep1* stationary state and bursting (Figure 6E).
3. Three ranges of multistability, each one supporting exactly two attracting regimes. There were two cases with three ranges of coexistence of exactly two attracting regimes (Figure 6F). These ranges supported the following types of coexistences: (1) the *dep2* and *dep1* stationary states, (2) bursting and the *dep1* stationary state, and (3) bursting and the *hyp1* stationary state. The case #1292494 in Figure 2C,D illustrates this scenario.
4. Three ranges of multistability, one of them involving tristability and the others bistability. All models with ranges of 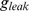 supporting tristability showed additional ranges supporting bistability. In 34 cases, there were two bistable ranges, one of them supported the coexistence of the *hyp1* and *dep1* stationary states, while the other supported the coexistence of the *dep1* stationary states and bursting (Figure 6G). Five cases exhibited ranges with bistability of the *hyp1* state and bursting, along with bistability of the *dep1* state and bursting (Figure 6H). Three cases exhibited two additional bistable ranges, both involving the *hyp1* and *dep1* stationary states, in addition to the range supporting tristability (Figure 6J).
5. Four ranges of multistability, one of them involving tristability and the others bistability. Along with a range supporting tristability, six cases exhibited a range supporting bistability of the *hyp1* and *dep1* stationary states, a range of coexistence of the *dep1* stationary state and bursting, as well as a range of coexistence of the *dep1* and *dep2* stationary states (Figure 6I).

**Figure 7:**
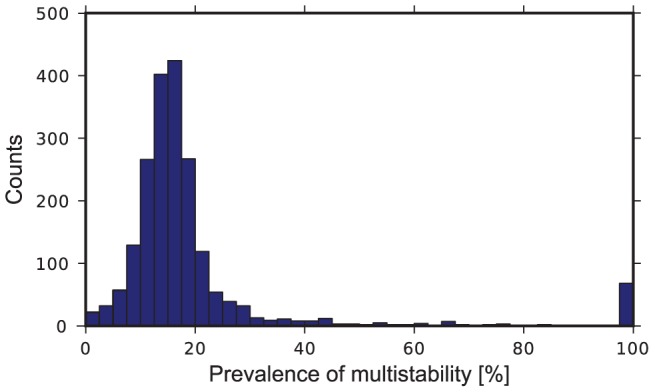
A histogram of prevalence of the multistability of the silent and bursting regimes. Prevalence of multistability was calculated as the percentage of the whole range of 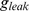 values supporting bursting that supports multistability of the stationary states and robust bursting.

In summary, tristability was found in 48 out of 2,223 unique robust bursting cases (2%). Adding up all multistable cases, we found that 2,016 cases (91% of the unique robust bursters) exhibited multistability under some range of 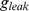 values. To assess the significance of these results we introduced a measure describing the sensitivity of multistability to variation of the leak conductance. For each case exhibiting multistability, we calculated the prevalence of multistability of bursting and silent regimes as the percentage of the whole range of 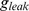 values supporting bursting that supported multistability of the stationary states and robust bursting. A histogram of the prevalence of the multistability showed a large peak around 17% (Figure 7). The histogram also had a smaller peak at 100%. Interestingly, all cases with tristability had the prevalence of 100%. The mean value of the prevalence of the multistability was 19.6% with the standard deviation 17.7%. If we drop outliers from consideration by excluding the cases with prevalence two times larger than the mean value, we obtain the mean value corresponding to the large peak. This adjusted mean value was 15.5% with standard deviation 5.6%. This analysis shows a high prevalence of multistability of bursting and silent regimes in the dynamics of the leech heart interneuron model.

### Multistability in the database

Besides determining the ranges supporting multistability for a wide range of 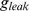 values, our methodology allowed us to check for its presence in any case extracted from the *robust burster* subset of the database, just by determining whether the original 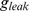 value belonged to the range supporting coexistence of bursting and other regimes. This value is marked in Figures 2B,D,F and 4B as a solid vertical blue line. For the case #1292494, the original 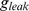 belonged to a range which supported the robust bursting as the only attractor available (indicated by the navy blue bar), thus no multistability was observed. For the case #637432 the original value of 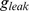 belonged to the range supporting multistability of bursting and *hyp1* (the range is indicated by the cyan bar, Figure 2E,F), while in the case #288298, the original 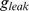
value belonged to the range supporting two regimes, bursting and *hyp2* stationary state (indicated by the green bar, Figure 2B). In the case #861497 the original 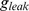
value belonged to a range supporting *hyp1*, *dep1* and bursting (indicated by a yellow bar) (Figure 4). Thus, for the last two cases, multistability was already present in the *robust burster* subset of the database.

Applying this procedure to all *robust burster* cases, out of the 2,387 models 421 (18%) were multistable for the original 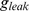 value in the database. This result is consistent with the results of the analysis of the prevalence of multistability of bursting and silent regimes. Out of those 421 cases, 361 exhibit bistability between bursting and the *hyp1* stationary state; 47 cases show bistability of bursting and the *dep1* stationary state; and one case shows bistability of bursting and the *hyp2* stationary state (Figure 6). Moreover, 12 cases demonstrated tristability: coexistence of bursting, the *hyp1* and *dep1* stationary states (Figure 4B and Figure 5).

### Functionality of half-center oscillators built from multistable neurons

The leech heart interneurons analyzed in the *robust burster* database are units of half-center oscillators (HCO) that control the leech heartbeat. The possibility of one of the cells going into a silent state presents a potentially dangerous situation for the leech. As stated previously, the analyzed cases show high prevalence of multistability of bursting and silence, with 89% of them exhibiting multistability for some range of 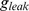
values. Hence, we developed the protocol described in the methods section to examine whether HCOs constructed of multistable neurons regain functional alternating bursting pattern after a perturbation setting one of the cells exactly into its stable rest state.

**Figure 8:**
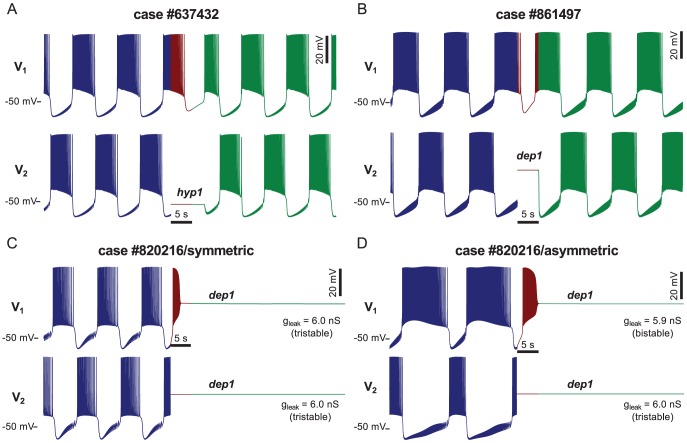
Perturbation of half-center oscillators constructed from pairs of multistable neurons. Functional alternating bursting pattern (blue) was interrupted by the perturbation, during which the synaptic interaction was blocked for 5 s (red) and one cell (bottom trace) was reset (not clamped) to its stable stationary state (one of the two states in the case of tristable cells). After the perturbation, the synaptic interactions were restored (green). Panels A–C show results for symmetric HCOs with two identical neurons. In the case #637432 neurons exhibit coexistence of the *hyp1* stationary state and bursting (A). In the cases #861497 (B) and #820216 (C) neurons are tristable (coexistence of the *hyp1* and *dep1* stationary states and bursting). Panel D shows an example of perturbation of an asymmetric HCO. One neuron (bistable, 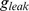 = 5.9 nS) differs in the leak conductance from the other neuron (tristable case as in C, 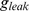 = 6.0 nS). The panels A and B represent majority of the cases by showing that an HCO regains functional bursting after perturbation. Panel C presents an example where the perturbation led to a dysfunctional activity. This dysfunctional outcome of the perturbation could be observed in asymmetric HCOs as well (D). The difference between the neurons' leak conductances is reflected in strong asymmetry in the bursting pattern before the perturbation.

In Figure 8, we present four examples: two with the functional pattern restored (A,B) and two with both cells caught in the depolarized rest states (C,D). Either bistable or tristable cases could be assembled into HCOs resistant to perturbation: a bistable case #637432 (Figure 8A) exhibiting coexistence of the *hyp1* stationary state and bursting (Figure 2E,F) and a tristable case #861497 (Figures 4,5,8B) demonstrating coexistence of the *hyp1* and *dep1* stationary states and bursting. For the first case (Figure 8A), when the synapses were blocked and the bottom cell set into the *hyp1* stationary state (red part of the time series), the top cell continued to burst according to its endogenous dynamics, since it was not being inhibited. When the synaptic connections were restored (green part of the time series), the upper cell was in the hyperpolarized phase of bursting, but already on its way towards initiation of the next burst. There were no synaptic currents to perturb the bottom cell towards its bursting regime, so it stayed close to the *hyp1* stationary state. When the top cell began its burst (still not inhibited by the bottom cell), the synaptic currents perturbed the bottom cell out of the basin of attraction of the stationary state, restoring its bursting activity and the functionality of the system as a half-center oscillator.

For the second case (Figure 8B), where two tristable cells formed the HCO, when the synaptic coupling was restored the top cell was already firing a burst due to its own dynamics. Thus, the bottom cell was immediately pushed out of the *dep1* stable stationary state into bursting, and the half-center oscillator recovered its alternating bursting pattern. Similar activity was obtained when we reset the cell to the *hyp1* stationary state, as in the bistable example (Figure 8A).

The half-center configuration was not always effective in restoring the functional pattern after this perturbation (Figure 8C). In the case #820216 a single neuron is tristable. It exhibits the *hyp1* and *dep1* rest states and bursting regime. In this example, both cells ended in the *dep1* depolarized stable rest state after the perturbation. The perturbation set the second cell to the *dep1* rest state during a burst. At the beginning of the perturbation the first cell was inhibited. Having the inhibition from the second cell removed the first cell experienced a rebound and ended in the depolarized rest state too. At the end of the perturbation both cells were found in their rest states and stayed there afterwards. This scenario was also observed in the case of an asymmetric HCO (Figure 8D). We modified the HCO from the previous example by changing 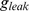 of one cell from 6 nS to 5.9 nS; the other cell remained at 6 nS. With this value the altered cell was bistable since the hyp1 stationary state lost stability via an Andronov-Hopf bifurcation at 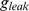 = 5.951 nS. This difference in the values between the leak conductance caused strong asymmetry of the bursting pattern but the perturbation still caused the switch into a dysfunctional silent regime.

To check whether the recovery of functionality was prevalent among other half-center oscillators built from multistable units, we explored the activity of HCOs constructed with the 421 cases that were multistable in the original database, as described above. Not all of those models displayed functional activity for the initial conditions given in [25], so our analysis was restricted to the 353 (84%) that were initially functional for at least one of the initial conditions. Applying the protocol to this subset, we observed that in 96% of the cases the HCOs recovered functional bursting after the perturbation, proving themselves robust against the presence of multistability involving stationary states. In the remaining 4% of cases, the HCOs were switched by perturbation into dysfunctional regimes where both cells were stuck in rest states, or at least one of them produced damped oscillations around a rest state, tonic spiking, or irregular bursting.

## Discussion

The same identified neurons and their synaptic connections in a circuit show a high level of variability from preparation to preparation and yet produce surprisingly similar, appropriate patterns of activity in accordance with their function [1]–[4]. This fact shifts the paradigm in modeling from searches for a canonical model, which would be perfectly tuned to experimental data, towards construction of populations of well-tuned cases of a model [24], [25], [32]. Application of a brute force database approach showed that, indeed, multiple sets of parameters produce a model exhibiting similar, functional activity, i.e., an activity with characteristics satisfying all constraints measured experimentally [20], [21]. With this study we demonstrate that multistability of oscillatory and silent regimes can be prevalent in dynamics of neuronal models and has to be taken into account.

A brute force database is a powerful tool for assessing and cataloging regimes of neuronal activity [2], [19]–[25], [33]. It sweeps through multidimensional parameter space, obtaining and categorizing the activities of the model for each parameter set, i.e., case. The goal is to describe the activity of each case with maximal completeness and store it into a database. Since the dynamics of neurons and networks can be multistable [6], [7], [10], [15]–[18] to achieve this goal one has to describe all possible regimes of activity. In the face of a large number of cases to be analyzed, which easily reaches an order of magnitude of 10 million, it is not feasible to use more than a few initial sets of state variables (initial conditions). This turns the investigation of multistability of the dynamics of each case into a computational challenge. This computational challenge raises a question: How common is multistability? If only a negligible number of models show multistability, it might appear that it is not worth the effort to investigate the database for multistability.

By using methods developed in the bifurcation theory, we extended an existing database of a leech heart interneuron, providing information on stationary states. To demonstrate a proof of these methods we analyzed a set of important cases in the database – the cases representing a single neuron which produces robust endogenous bursting when isolated and functional bursting when assembled into a half-center oscillator. For each of these cases we upgraded the database with entries describing all stationary states found, stable and unstable. The number of stationary states varied from case to case from 1 to 5. This analysis also provided new information on possible regimes of activity. We found that the model could have excitability block, represented by stable depolarized stationary states, at two different levels of membrane potential. The highly depolarized one occurs around +20 mV and is reported here for the first time.

A high percentage of the cases considered showed multistability of activity regimes. We found that 18% of the cases exhibited the coexistence of stationary states and bursting. Furthermore, considering these cases under leak conductance variation, 91% of them exhibited multistability in some range of leak conductance. These results complement studies which have established the prominent role of leak currents as a target of modulation [34]–[36]. The results show that modulation of the leak current can lead not only to transitions between silent, bursting and spiking regimes but also into and out of the multistable dynamics.

The prevalence of multistable cases in the database of a leech heart interneuron is intriguing since these neurons pace an animal's heartbeat, and multistability of an oscillatory regime and a stationary state poses a threat to the functionality of the central pattern generator, which is required to produce a persistent, steady pattern. These results raise questions concerning how a neuronal network exhibiting co-existence of functional and dysfunctional regimes can maintain normal operation in the face of naturally occurring variation in parameters and external perturbations.

We suggest that the half-center oscillator configuration could serve as a network-based protective mechanism against instances of dysfunctional multistability. By analyzing multistable models coupled via mutual inhibition, we verified that in the majority (96%) of the robust bursting cases, the functional regime is robust against tested types of perturbations of initial conditions and synaptic coupling, showing that multistability involving stationary states is not necessarily disruptive to HCO functional bursting activity. This conclusion is consistent with our previous findings that half-center configuration brings robustness to the CPG against deviations in the dynamics of the single neurons involved [10].

The main results presented here were obtained for symmetrical half-center oscillators with two identical neurons. The alternating bursting pattern was sensitive to differences in properties of the two cells, exhibiting a difference between the burst durations of the two cells (Figure 8D). It seems obvious that there exist no two identical neurons in living neuronal networks. This notion leads us to further questions concerning homeostatic mechanisms preserving functional patterns under variation of cellular properties and persistence of the dysfunctional regimes [37], [38]. In other systems of coupled endogenously bursting cells, heterogeneity of cellular properties in the network was shown to eliminate some dynamical regimes. This effect is particularly prominent in cases of electrically coupled cells like the β-cells of the islet of Langerhans [39]. In our model of the leech heartbeat HCO, this mechanism does not eliminate the high risks of the multistability with rest states (Figure 8D). The rest states observed in single cells are preserved in HCOs, if silent cells cannot interact. If a perturbation places both cells into their stable rest states without the possibility of synaptic interaction the HCO would remain silent. This consideration applies to asymmetric HCOs as well. Here we considered a perturbation less severe but one more likely to happen in a real system: only one cell was set into a stable rest state. Although most cases of the leech heartbeat HCO model restored functional pattern, 4% cases ended in a dysfunctional regime. One could observe that either a symmetrical or heterogeneous HCO can get trapped in a silent regime (Figure 8CD).

In our previous studies, the canonical models of a leech heart interneuron and of a HCO were instrumental in explaining experimental results and showing predictive power for new experiments [10], [26]. These models capture the electrical activity of an isolated interneuron and of a HCO under a variety of experimental conditions [10], [26], [40]–[47]. They were developed following the Hodgkin-Huxley formalism, and have incorporated the kinetics of currents measured in voltage-clamp experiments. These models provide a solid framework for studies of the variability of cellular properties among animals. As these models have been so valuable as a predictive tool in the past [10], [44], [47], we anticipate that the predictions made with the brute-force database of these models are credible. Our results suggest that it would be much easier to detect the multistability in the pharmacologically isolated neurons first, since the half-center motifs of the CPG circuit can restore functional bursting pattern in the face of potentially disruptive multistability. We predict that multistability of bursting and silent regimes can be demonstrated experimentally by increasing the leak conductance using a dynamic clamp technique [10]. To achieve this demonstration, we would have to solve a number of technical issues: the dynamic clamp requires intracellular recording with sharp microelectrodes. Such electrodes add significant leak. When applied to the leech heart interneurons, these electrodes transform the regime of neuronal activity from endogenously bursting into endogenously tonically spiking. In future we plan to identify modifications of ionic and leak currents (using Dynamic Clamp, e.g.), which would allow us to record endogenously bursting and silent regimes of the leech heart interneurons while recording intracellularly. Having this milestone achieved, we anticipate detecting multistability of the bursting and silent regimes within the border region between the transitions from silence into bursting and from bursting into silence. On the other hand, multistability could play unknown important roles in normal functioning of the leech heart CPG.

### Multifunctional central pattern generators

Multistability of single neurons could be a valuable requisite property for a variety of functions executed by neuronal networks. Multistable neurons are a substrate for memory units, toggle switches, and elements of multifunctional central pattern generators. A multifunctional central pattern generator is a neuronal network which can centrally generate more than one functional rhythmic motor pattern [48]–[56]. Studies of multifunctional CPGs in invertebrate preparations benefit from multiple advantages: the neuronal circuits can be described in terms of identified neurons and the connections between them. For example, in the medicinal leech there is a set of interneurons contributing to either a slow pattern, producing crawling, or a fast pattern, producing swimming. In the jellyfish *Aglantha digitale* multistability in the dynamics of single neurons is implicated in mechanisms underlying two different behaviors. This jellyfish shows two different swimming patterns: it swims slowly when feeding and rapidly when escaping [48]. These two different modes of swimming are driven by motoneurons and are explained by the fact that these motoneurons exhibit two regimes of spike propagation, a slow one based on T-type calcium current and a fast one driven by sodium current. The mechanisms of multistability are generally not well understood, however.

### Mechanisms of multistability

The mechanisms underlying multistability can be thoroughly studied by applying the theory of dynamical systems [5], [6]–[8], [11], [16], [17], [54], [57]–[63]. A key ingredient of a description of a mechanism is the identification of the unstable regime(s) which creates the boundary separating observable regimes. Among such unstable regimes leading to multistability, the most ubiquitous are saddle stationary states and saddle periodic orbits [60]. Using Morris- Lecar model as an example, Rinzel and Ermentrout showed two different mechanisms of bistability of spiking and silence [60]. In one case the stable manifold of a saddle stationary state separated a stable stationary state from limit cycle representing spiking activity, and in the other case the saddle periodic orbit separated these regimes [60]. In a mechanism described by Rinzel, an unstable periodic orbit representing unstable sub-threshold oscillations is responsible for the co-existence of periodic tonic spiking and silent regimes [5], [6]. The stable manifold of the periodic orbit separates silent and periodic spiking regimes [5]. Even a simple model of a single neuron can show a variety of different mechanisms underlying the coexistence of silence, subthreshold oscillations, spiking, and bursting in different combinations. In our previous work, we described six different types of multistability in a single simplified model [18]. Information on the bifurcations limiting the unstable regime is also important for understanding multistability in the neuronal dynamics and for description of the mechanisms of multistability [5], [6], [16]–[18], [60], [61]. Rinzel showed mechanisms of multistability which involve fold bifurcations for the stationary states and periodic orbits, homoclinic bifurcation, and torus bifurcation [61], [62]. The slow-fast decomposition techniques are instrumental in the analysis of the mechanisms supporting multistability [60]–[63]. They allow thorough description of multistability in terms of the averaged dynamics of the slow variables [2], [6], [11], [40], [41], [58]–[63]. The methods described here allow one to determine stationary states of the full model, stable and unstable, and to conduct large scale screening of a model database for new mechanisms of multistability, stationary states and other observed regimes. The brute-force database approach is a promising tool for screening cases of a model for novel mechanisms supporting multistability.
